# Oxygenated polycyclic aromatic hydrocarbons from ambient particulate matter induce electrophysiological instability in cardiomyocytes

**DOI:** 10.1186/s12989-020-00351-5

**Published:** 2020-06-11

**Authors:** Sujin Ju, Leejin Lim, Han-Yi Jiao, Seok Choi, Jae Yeoul Jun, Young-Jae Ki, Dong-Hyun Choi, Ji yi Lee, Heesang Song

**Affiliations:** 1 Department of Biomaterials, Chosun University Graduate School, Gwangju, 61452 South Korea; 2 Cancer mutation Research Center, Chosun University, Gwangju, 61452 South Korea; 3 Department of Physiology, Chosun University School of Medicine, Gwangju, 61452 South Korea; 4 Department of Internal Medicine, Chosun University School of Medicine, Gwangju, 61452 South Korea; 5 Department of Environmental Science and Engineerings, Ewha Womans University, Seoul, 03760 South Korea; 6 Department of Biochemistry and Molecular Biology, Chosun University School of Medicine, Gwangju, 61452 South Korea

**Keywords:** Ambient particulate matter, Oxygenated polycyclic aromatic hydrocarbons, Electrophysiological instability, Cardiomyocytes, Reactive oxygen species

## Abstract

**Background:**

Epidemiologic studies have suggested that elevated concentrations of particulate matter (PM) are strongly associated with an increased risk of developing cardiovascular diseases, including arrhythmia. However, the cellular and molecular mechanisms by which PM exposure causes arrhythmia and the component that is mainly responsible for this adverse effect remains to be established. In this study, the arrhythmogenicity of mobilized organic matter from two different types of PM collected during summer (SPM) and winter (WPM) seasons in the Seoul metropolitan area was evaluated. In addition, differential effects between polycyclic aromatic hydrocarbons (PAHs) and oxygenated PAHs (oxy-PAHs) on the induction of electrophysiological instability were examined.

**Results:**

We extracted the bioavailable organic contents of ambient PM, measuring 10 μm or less in diameter, collected from the Seoul metropolitan area using a high-volume air sampler. Significant alterations in all factors tested for association with electrophysiological instability, such as intracellular Ca^2+^ levels, reactive oxygen species (ROS) generation, and mRNA levels of the Ca^2+^-regulating proteins, sarcoplasmic reticulum Ca^2+^ATPase (SERCA2a), Ca^2+^/calmodulin-dependent protein kinase II (CaMK II), and ryanodine receptor 2 (RyR2) were observed in cardiomyocytes treated with PM. Moreover, the alterations were higher in WPM-treated cardiomyocytes than in SPM-treated cardiomyocytes. Three-fold more oxy-PAH concentrations were observed in WPM than SPM. As expected, electrophysiological instability was induced higher in oxy-PAHs (9,10-anthraquinone, AQ or 7,12-benz(a) anthraquinone, BAQ)-treated cardiomyocytes than in PAHs (anthracene, ANT or benz(a) anthracene, BaA)-treated cardiomyocytes; oxy-PAHs infusion of cells mediated by aryl hydrocarbon receptor (AhR) was faster than PAHs infusion. In addition, ROS formation and expression of calcium-related genes were markedly more altered in cells treated with oxy-PAHs compared to those treated with PAHs.

**Conclusions:**

The concentrations of oxy-PAHs in PM were found to be higher in winter than in summer, which might lead to greater electrophysiological instability through the ROS generation and disruption of calcium regulation.

## Background

Exposure to ambient particulate matter (PM) is associated with increased cardiovascular morbidity and mortality. After revealing the association between PM exposure and the causative risks involved in all mortality cases in the US [1], various epidemiological and experimental studies have reported that elevated PM concentrations were closely associated with increase in cardiovascular diseases (CVD), including myocardial infarction, stroke, arrhythmia, and venous thromboembolism [2–4]. In addition, epidemiological studies have shown a positive correlation between elevated levels of PM and the incidence of life-threatening ventricular arrhythmias [5, 6]. However, most previous studies have only focused on revealing epidermiological correlations between air pollution and the prevalence of CVD [7, 8], especially arrhythmia, although few other studies emphasized on the underlying mechanisms in cardiomyocytes [9]. Indeed, experimental studies have suggested that PM exposure increases cardiac oxidative stress and electrophysiological changes in rats [10, 11]. In addition, Kim et al. demonstrated that arrhythmic parameters, such as action potential duration (APD), early afterdepolarization (EAD), and ventricular tachycardia (VT), were significantly increased in diesel exhausted particle (DEP)-infused rat hearts due to oxidative stress and calcium kinase II activation [9].

Ambient PM, composed natural and anthropogenic particles, is a complex mixture of organic and inorganic compounds [12]. In particular, there is growing evidence that polycyclic aromatic hydrocarbons (PAHs) and their oxygenated derivatives (oxy-PAHs), which are major organic components of ambient PM, play an important role in the correlation between air pollution and increased cardiovascular morbidity and mortality rates [13–15]. PAHs and oxy-PAHs are found in cigarette smoke and are generated by different combustion processes in urban environments; the sources of PAHs and oxy-PAHs include motor vehicles, residential heating, fossil fuel combustion in energy and industrial processes, and municipal and medical incinerators [16, 17]. In addition, oxy-PAHs also originate from reactions between PAHs and hydroxyl radicals, nitrate radicals, other organic and inorganic radicals, and ozone [18], or from photo-oxidation of PAHs by singlet molecular oxygen [19]. The carcinogenic potential of various PAHs, which may act as major contributors to the mutagenic activity of ambient PM, have been reported [20, 21]. Moreover, it has been demonstrated that oxy-PAHs have the highest human-cell mutagenic potential of all respirable airborne particles in the northeastern United States [21]. In addition, because of their ability to oxidize nucleic acids, proteins, and lipids, oxy-PAHs might also induce severe redox stress in cells and tissues [3–5]. Therefore, we hypothesize that oxy-PAHs induce more severe arrhythmia than PAHs via oxidative stress. To test this hypothesis and identify the underlying mechanisms of oxy-PAHs induced arrhythmia, we compared seasonal concentrations of PAHs and oxy-PAHs and the amount of oxidative stress induced by these compounds in cardiomyocytes. Further, we determined the levels of ROS and electrophysiological alterations caused by selected PAHs and oxy-PAHs.

## Results

### Ambient particles promotes electrophysiological instability

**Fig. 1:**
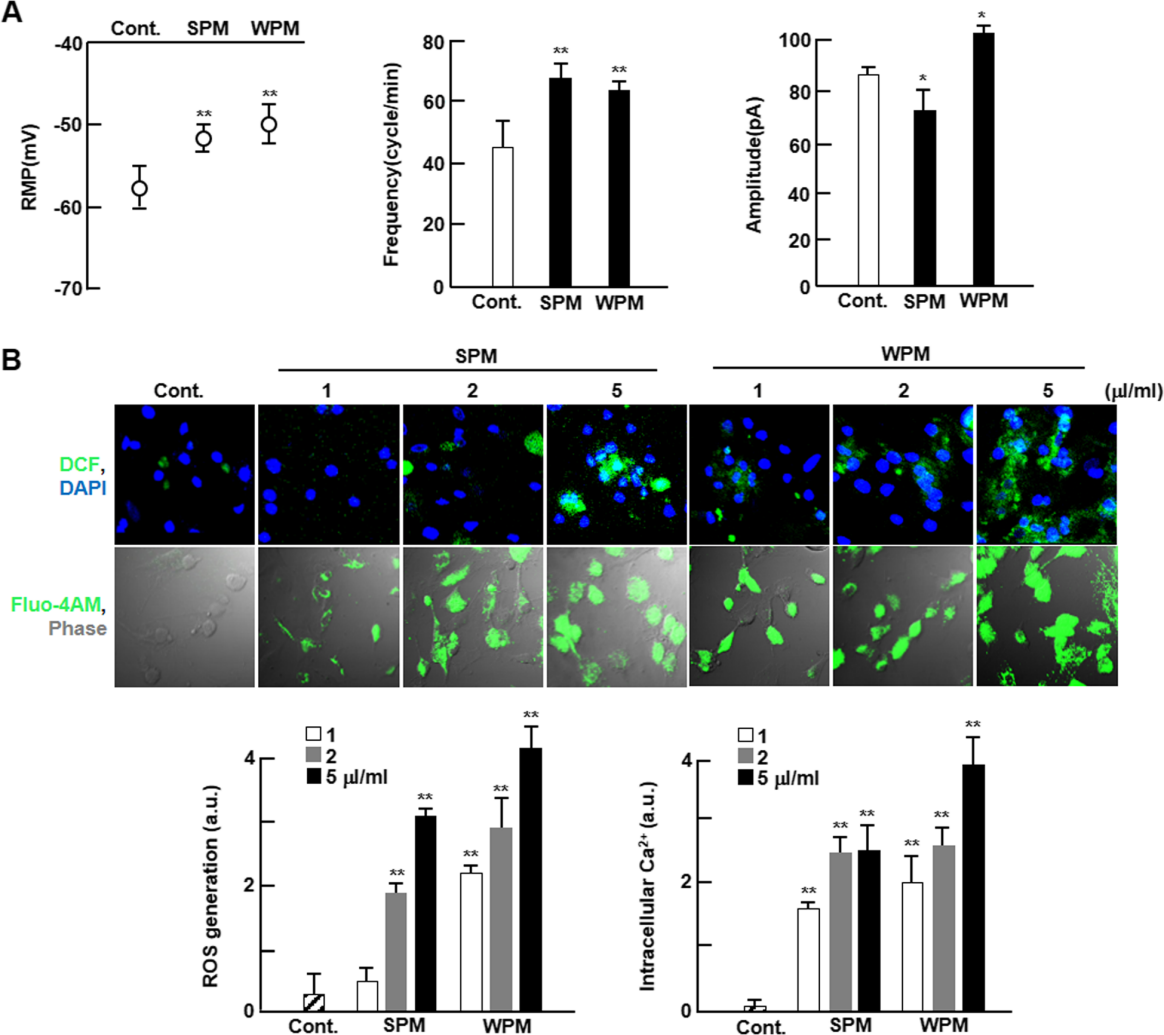
Effect of ambient PM on the electrophysiological stability of cardiomyocytes. **a** Electrophysiological parameter data averages from cardiomyocytes with ambient PM infusion (*n* = 6). **b** Representative fluorescence images of ROS generation or intracellular calcium in PM-treated cardiomyocytes using H_2_DCF-DA (top, green) or Fluo-4 AM (under, green), respectively (*n* = 5). Nuclei were stained with DAPI (blue). The rate of fluorescence in every case was quantified by SIBIA software. Values are represented as mean ± SD. *P < 0.05 or **P < 0.01 compared with control. RMP: resting membrane potential, SPM: PM collected in summer, WPM: PM collected in winter

To investigate electrophysiological alterations caused by ambient PM, we analyzed the action potential parameters using a patch clamp system. As shown in Fig. 1a, ambient PM rapidly increased the action potential (AP) frequency, depolarized the resting membrane potential (RMP), and reduced the action potential amplitude (APA). Importantly, ambient PM increased the action potential duration (APD) for both 50 and 90% repolarization (APD_50_ and APD_90_). We observed that APD increased immediately after switching to PM-containing solution; it increased with time and reached a steady state within 5 min. The induced electrophysiological instability was remarkably higher in WPM-treated cardiomyocytes than in SPM-treated cardiomyocytes. We then investigated the ROS generation and subsequent intracellular Ca^2+^ disturbance by ambient PM using a fluorescence assay. As seen in Fig. 1c, ROS generation was significantly increased in a dose-dependent manner in cardiomyocytes treated with ambient PM collected in both summer and winter. The increase in ROS generation was greater in WPM-treated cardiomyocytes than in SPM-treated cardiomyocytes. Intracellular Ca^2+^ contents showed a similar trend as ROS generation (Fig. 1c).

### Ambient particles regulate expression of Ca^2+^-related genes and the dependent signaling pathways

**Fig. 2:**
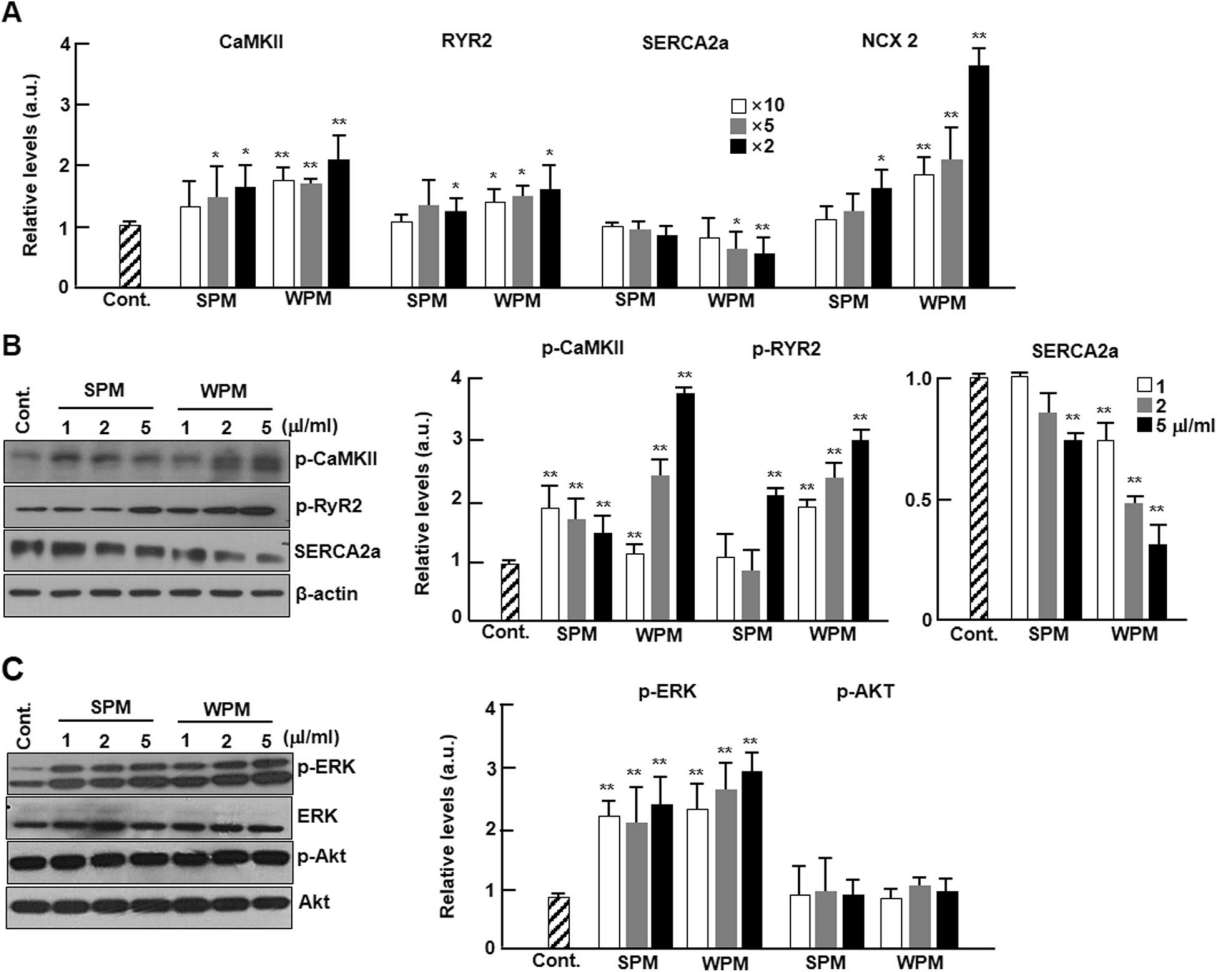
Effect of ambient PM on expression of Ca^2+^-related genes and dependent signals in cardiomyocytes. Cardiomyocytes were treated with control, SPM, or WPM at the indicated concentrations for 24 h and harvested for RNA extraction and western blotting. **a** mRNA levels of CaMKII, RyR2, SERCA2a, and NCX2 were quantified by qPCR (*n* = 4). Gene expression was normalized to GAPDH. **b** The expression of phospho-CaMKII, phospho-RyR2, and SERCA2a were analyzed by western blotting (*n* = 5). Protein levels were normalized against β-actin. **c** The expression levels of phosphorylated ERK1/2 and Akt were analyzed by western blotting (*n* = 4). The protein levels were normalized to total ERK or Akt levels, respectively. All values are represented as mean ± SD. *P < 0.05 or **P < 0.01 compared with control

The effects of ambient PM on Ca^2+^ related genes in cardiomyocytes were investigated. The contraction of cardiomyocytes is triggered by the influx of Ca^2+^ into the cytosol through voltage-gated L-type Ca^2+^ channels, and this influx initiates the release of Ca^2+^ from the sarcoplasmic reticulum (SR) via ryanodine receptor 2 (RyR2). For relaxation, there is a rapid Ca^2+^ reuptake into the SR through SR Ca^2+^-ATPase (SERCA2a) and extrusion via Na^+^/Ca^2+^-exchanger (NCX). Calmodulin kinase II (CaMKII) phosphorylates RyR2 to enhance SR Ca^2+^ release [22]. mRNA expression levels of CaMKII and RyR2 increased in a dose-dependent manner in cardiomyocytes treated with both SPM and WPM. SERCA2a was significantly decreased in cardiomyocytes treated with WPM but not with SPM. Interestingly, we evaluated the altered levels of all NCX isoforms, NCX1, NCX2, and NCX3, and identified that only the levels of NCX2 mRNA were altered and this chage was only observed at the highest concentration of SPM-treated cardiomyocytes and at all concentrations of WPM-treated cardiomyocytes (Fig. 2a). The data for NCX1 and 3 are not shown. We observed that the phosphorylated levels of CaMKII and RyR2 and the protein expression levels of SERCA2a were significantly altered in a similar manner as the mRNA expression levels, demonstrating that the ROS generated by ambient PM not only affected the expression but also the activity of calcium-regulating genes in cardiomyocytes (Fig. 2b). Furthermore, the levels of phosphorylated ERK dramatically increased in ambient particle-treated cardiomyocytes, but the levels of phosphorylated Akt did not appear to be altered (Fig. 2c).

### Scavenging of ROS by NAC attenuates the electrophysiological instability due to ambient particles

**Fig. 3:**
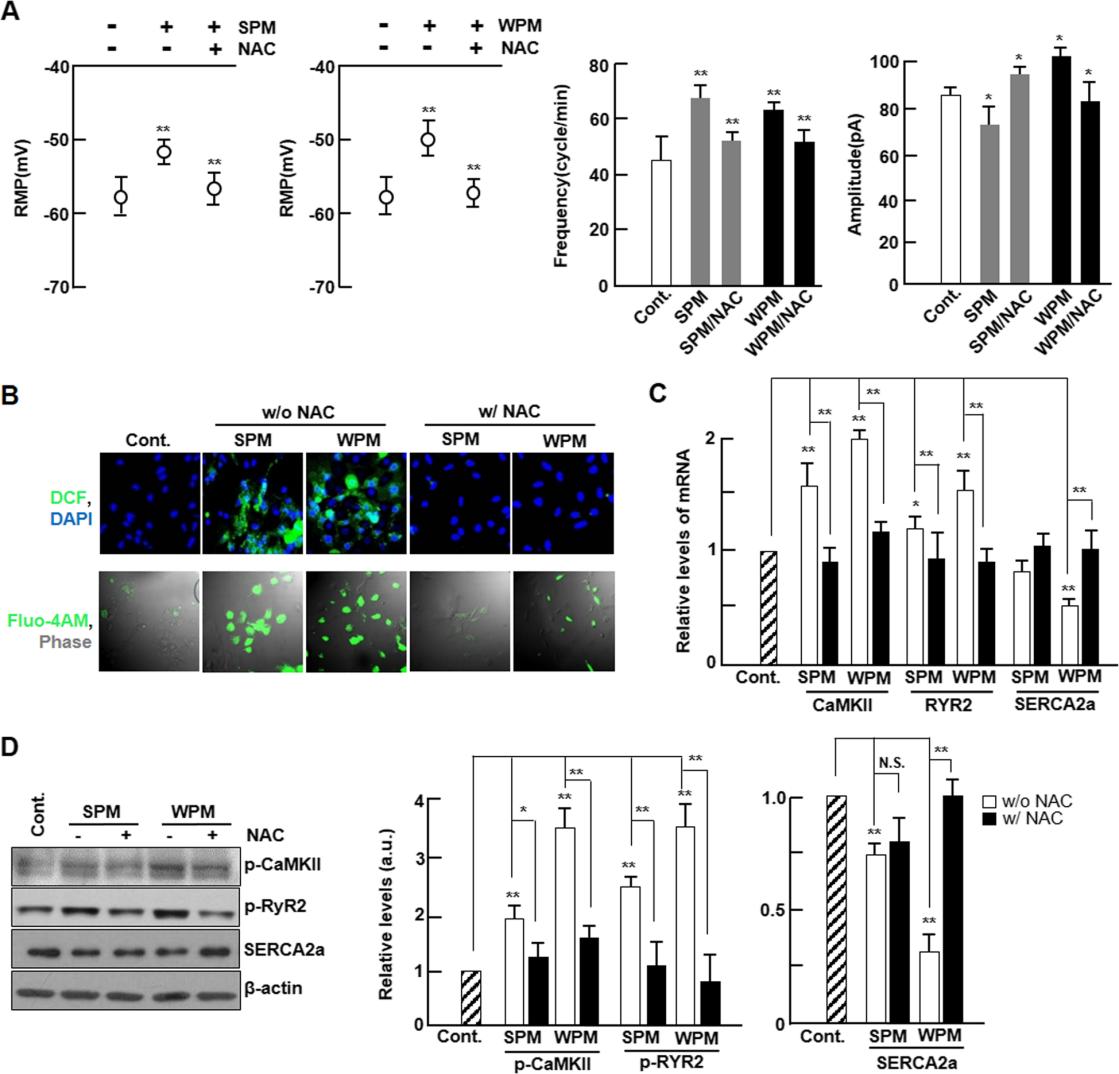
Effect of an ROS scavenger on the electrophysiological stability of cardiomyocytes. **a** Electrophysiological parameter data averages from cardiomyocytes with ambient PM infusion with or without NAC (*n* = 4). **b** Representative fluorescence images of ROS generation or intracellular calcium in PM-treated cardiomyocytes with or without NAC using H_2_DCF-DA (top, green) or Fluo-4 AM (under, green), respectively (*n* = 4). Nuclei were stained with DAPI (blue). The rate of fluorescence for every sample was quantified by SIBIA software. **c** The mRNA levels of CaMKII, RyR2, and SERCA2a in the absence or presence of NAC were quantified by qPCR (*n* = 4). Gene expression levels were normalized to GAPDH. **d** The expression levels of phospho-CaMKII, phospho-RyR2, and SERCA2a in the absence or presence of NAC were analyzed by western blotting (*n* = 5). Protein levels were normalized to β-actin. Values are represented as mean ± SD. *P < 0.05 or **P < 0.01 compared with non-treated controls

To confirm that the induction of electrophysiological instability in cardiomyocytes by ambient PM was specifically due to ROS, we investigated the effects of ROS scavenger, N-acetyl cysteine (NAC), on electrophysiological alterations caused by ambient PM using a patch clamp system. As shown in Fig. 3a, treatment with NAC resulted in a significant improvement in AP frequency, depolarized RMP, APA, and APD_50_ and APD_90_ values. We observed that ROS generation and intracellular Ca^2+^ contents were successfully attenuated in PM-treated cardiomyocytes by NAC treatment (Fig. 3b and c). In addition, alterations in mRNA or protein expression levels of CaMKII, RyR2, and SERCA2a by PM were rescued by NAC treatment (Fig. 3d and e).

### PAHs and oxy-PAHs result in differential ROS generation and Ca^2+^ perturbations

**Fig. 4:**
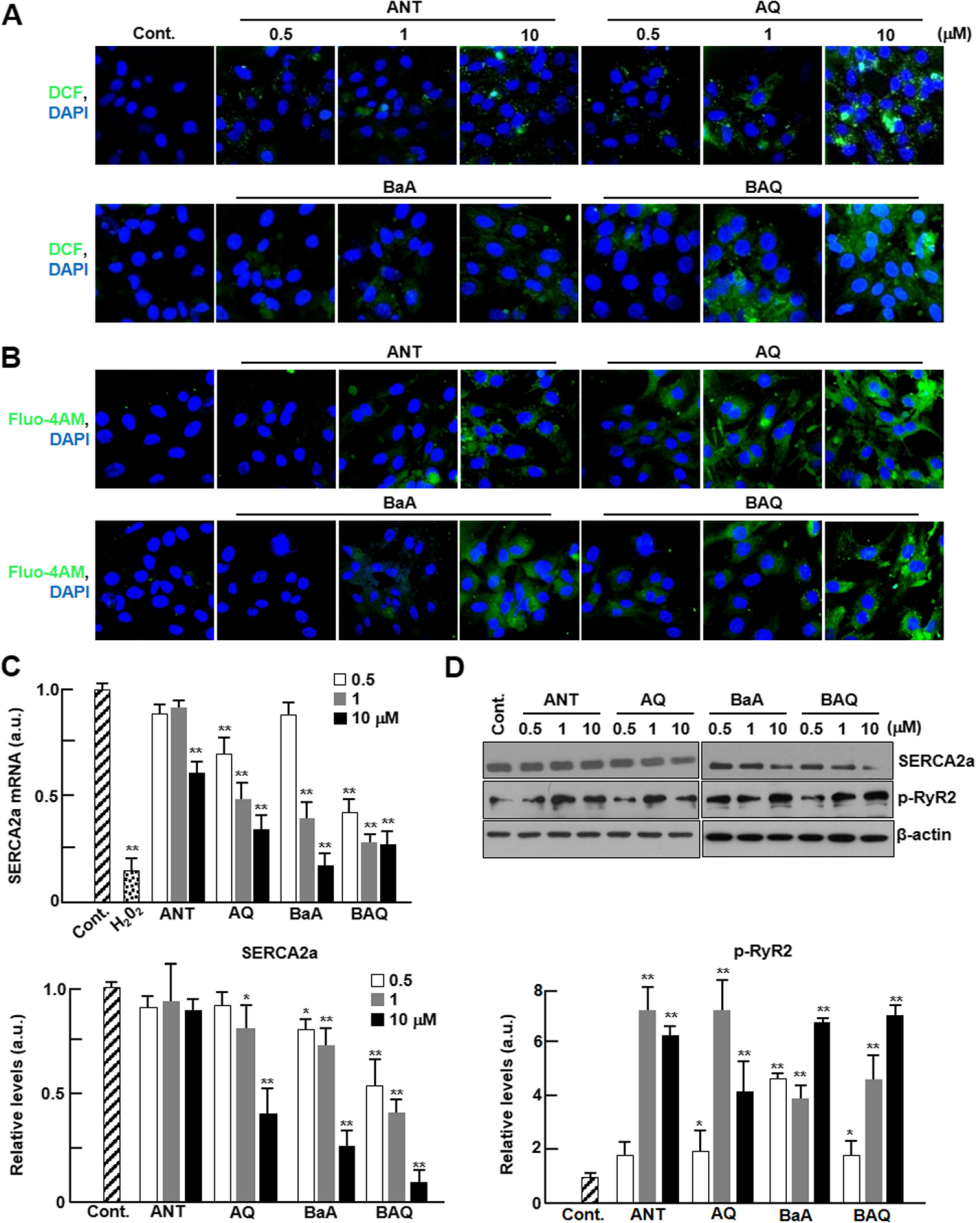
Effect of PAH and oxy-PAH on ROS generation and Ca^2+^ perturbation. Cardiomyocytes were treated with control (DMSO), ANT, AQ, BaA, or BAQ at the indicated concentrations and analyzed for ROS generation, intracellular Ca^2+^ levels, and expression of Ca^2+^-related genes. Representative fluorescence images of cardiomyocytes loaded with (**a**) H_2_DCF-DA (green) and (**b**) Fluo-4 AM (green) (*n* = 4). Nuclei were stained with DAPI (blue). (**c**) The mRNA levels of SERCA2a were quantified by qPCR (*n* = 4). Gene expression levels were normalized to GAPDH and H_2_O_2_ (200 nM) was used as a positive control. **d** The expression levels of phospho-CaMKII and SERCA2a were analyzed by western blotting (*n* = 5). Protein levels were normalized to β-actin. All values are represented as mean ± SD. *P < 0.05 or **P < 0.01 compared with control

Ambient particles collected in the Seoul metropolitan area contain organic matter, such as PAHs and oxy-PAHs, that might act as key mediators of ROS generation. As shown in Table 1, both PAHs and oxy-PAHs were contained with higher concentrations in WPM than SPM. In addition, we presented a standardized ratio of individual PAHs and oxy-PAHs mobilized from PM to organic carbon (OC) concentrations (Table 1). As shown in Table 1, ratio of PAHs and oxy-PAHs in WPM were 3–5 fold greater than those in SPM even though OC concentration in SPM and WPM are similar. We, therefore, hypothesized that PAHs and their oxygenated derivatives are the main components of ambient PM that induce electrophysiological instability in cardiomyocytes. We investigated the effects of the two kinds of PAHs (Table 2), anthracene (ANT) and benz(a) anthracene (BaA), and their oxygenated derivatives, 9,10-anthraquinone (AQ) and 7,12-benz(a) anthraquinone (BAQ), on ROS generation and Ca^2+^ perturbation in cardiomyocytes. Concentrations of 0.5, 1, and 10 μM PAHs or oxy-PAHs were used in this test and were shown to have no significant effects on cardiomyocyte viability (data not shown). As shown in Fig. 4a and b, ROS generation and intracellular Ca^2+^ contents significantly increased in cardiomyocytes treated with each PAH and oxy-PAH in a dose-dependent manner. As expected, we observed that the alterations were notably greater in cardiomyocytes treated with oxy-PAHs than in those treated with PAHs.

**Table 1:** Concentrations of PAHs and oxy-PAHs with ratio of composition in OC of ambient PM10

Organic compounds	SPM			WPM		
	ng/m^3,a)^	ng/mL^b)^	(ng/μg)*100^c)^	ng/m^3^	ng/mL	(ng/μg)*100
**PAHs**						
Phenanthrene	0.09	0.36	1.7	2.44	9.84	31.2
Anthracene	–	–	0.0	0.23	0.93	2.9
Fluoranthene	0.23	0.93	4.4	2.78	11.21	35.4
Pyrene	0.23	0.93	4.4	2.03	8.18	26.0
Retene	–	–	0.0	1.31	5.28	16.7
Benz [a]anthracene	0.24	0.97	4.7	0.98	3.95	12.5
Chrysene	0.17	0.69	3.2	0.90	3.63	11.5
Benzo [b]fluoranthene	0.41	1.65	7.8	1.41	5.69	18.0
Benzo [k]fluoranthene	0.25	1.01	4.9	1.16	4.68	14.8
Benzo [e]pyrene	0.20	0.81	3.9	0.93	3.75	11.9
Benzo [a]pyrene	0.21	0.85	4.1	1.00	4.03	12.7
Indeno[1,2,3-cd]fluoranthene	0.04	0.16	0.8	0.39	1.57	5.0
Dibenz [a,h]anthracene	0.05	0.20	0.9	0.47	1.90	6.0
Indeno[1,2,3-cd]pyrene	0.17	0.69	3.3	1.43	5.77	18.3
Benzoperylene	0.16	0.65	3.0	1.07	4.31	13.7
Coronene	0.07	0.28	1.4	0.80	3.23	10.3
Total	2.53	10.21	48.5	19.32	77.89	246.9
**Oxy-PAHs**						
1,4-Naphthalenedione	0.47	1.90	8.0	1.12	4.52	18.1
9,10-Anthracenedione	0.37	1.49	7.7	1.37	5.52	17.3
9-Fluorenone	0.73	2.94	18.1	0.67	2.70	8.7
Perinaphthenone	–	–	–	2.38	9.60	28.0
Xanthone	–	–		0.47	1.90	5.5
5,12-Naphthacenedione	–	–	–	–	–	–
Benz [a]anthracene-1,12-dione	**–**	–	–	0.79	3.19	10.6
Total	1.57	6.33	33.8	6.80	27.42	88.2
**Carbon species**						
Organic Carbon (OC)	5.20	20.97		7.83	31.57	
Elementary Carbon (EC)	1.82	7.34		1.96	790	
Water Soluble OC (WSOC)	2.20	8.87		3.56	14.35	

SPM and WPM are particulate matters collected in the Seoul metropolitan area during summer and winter seasons, respectively

^a)^ Ambient concentrations of individual compounds

^b)^ Injected concentrations to cells

^c)^ Ratio of individual compounds in OC

**Table 2:** Characteristics of PAHs and oxy-PAHs used in this study

Compound	Abbreviated name	Structure	Molecular formula	Molecular weight
**PAHs**				
Anthracene	ANT		C_14_H_10_	178.22
Benz(a)anthracene	BaA		C_18_H_12_	228.28
**Oxy-PAHs**				
9,10-Anthraquinone	AQ		C_14_H_8_O_2_	208.21
7,12-Benz(a)anthraquinone	BAQ		C_18_H_10_O_2_	258.27

**Fig. 5:**
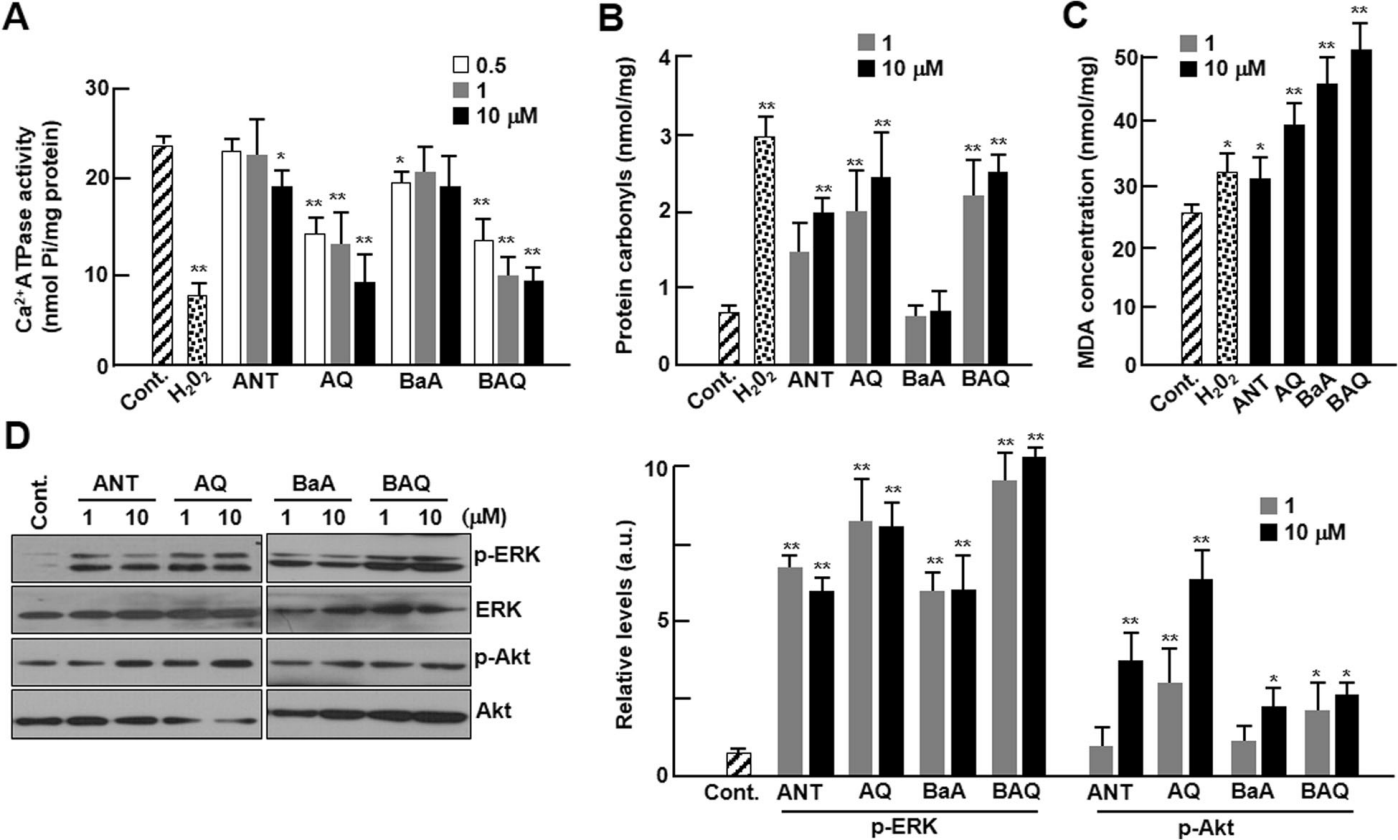
Biochemical effects of PAHs and oxy-PAHs. Cardiomyocytes were treated with control (DMSO), ANT, AQ, BaA, or BAQ at the indicated concentrations for 24 h. **a** Ca^2+^-dependent ATPase activity was assessed by measuring the quantity of inorganic phosphate (Pi) (*n* = 4). **b** Protein oxidation was assayed by measuring carbonyl formation (*n* = 4). **c** MDA concentration was assessed by measuring TBARS and normalized to the amounts of proteins (*n* = 4). **d** The expression levels of phosphorylated ERK1/2 and Akt were analyzed by western blotting (*n* = 4) and the protein levels were normalized to total ERK or Akt levels, respectively. H_2_O_2_ (200 nM) was used as a positive control. All values are represented as mean ± SD. *P < 0.05 or **P < 0.01 compared with control

mRNA expression of SERCA2a significantly decreased only in cardiomyocytes treated with 10 μM ANT and 1 and 10 μM of BaA, but the expression levels significantly decreased in cardiomyocytes treated with all concentrations of oxy-PAHs, AQ, and BAQ (Fig. 4c). The protein expression levels of SERCA2a also significantly decreased in cardiomyocytes treated with all concentrations of BAQ. However, there were no alterations in ANT-treated cardiomyocytes and a significant decrease was observed only in 10 μM AQ and BaA-treated cells (Fig. 4d). Interestingly, SERCA2a activities were significantly decreased by at least 2-fold for all concentrations of oxy-PAHs, AQ, and BAQ, but no changes were observed for PAHs, ANT, and BaA (Fig. 5a). It has been known that intracellular ROS might catalyze protein carbonylation [23] and malondialdehyde (MDA) formation [24]. Protein carbonylation was significantly increased in the H_2_O_2_-treated positive controls (Fig. 5b). We did not observe protein carbonylation in BaA-treated cardiomyocytes, whereas there was a significant increase in oxy-PAHs-, AQ-, and BAQ-treated cells. There was also a significant increase in protein carbonylation level in ANT-treated cardiomyocytes, but the increase was lower than in cells treated with oxy-PAHs (Fig. 5b). MDA formation was also increased in all samples, and the increase was greater in oxy-PAHs- than in PAHs-treated cardiomyocytes (Fig. 5c). Furthermore, phosphorylated ERK levels were dramatically increased in cardiomyocytes treated with each of the four PAHs, but this increase was greater in cardiomyocytes treated with oxy-PAHs than in those treated with PAHs (Fig. 5d). Phosphorylated Akt increased only when the cells were treated with 10 μM of ANT and all concentrations of AQ (Fig. 5d).

### The aryl hydrocarbon receptor mediates cytotoxicity of PAH and oxy-PAH

**Fig. 6:**
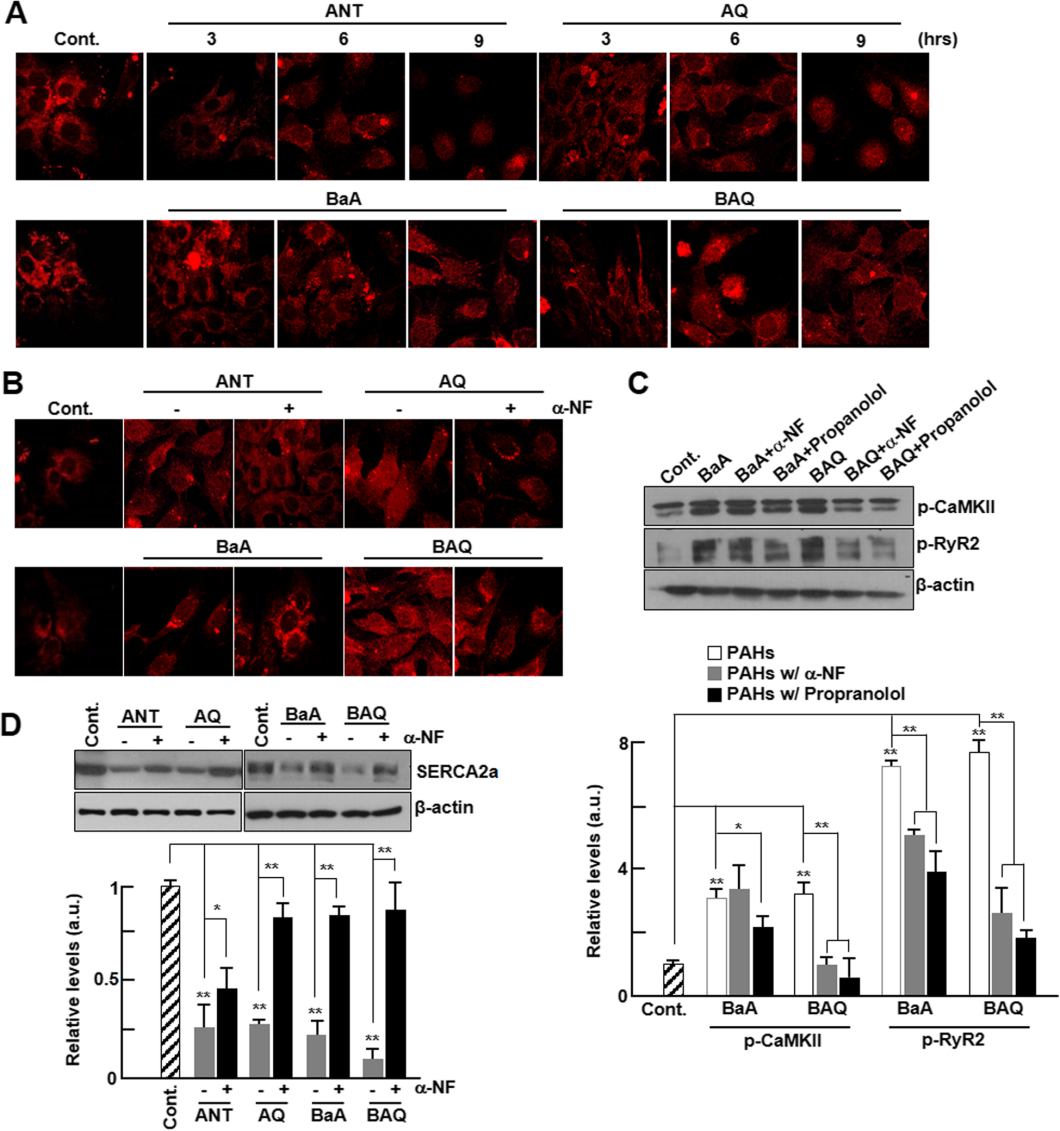
Nuclear translocation of PAHs and oxy-PAHs by AhR. **a** Cardiomyocytes were treated with control (DMSO), 10 μM of ANT, AQ, BaA, or BAQ and stained at the indicated time points with anti-AhR antibodies (red). Representative fluorescence images show the translocation of AhR in the nucleus. Scale bar, 400 μm. **b** Representative fluorescence images of cardiomyocytes with or without α-NF. The cells were stained with anti-AhR antibodies (red). **c** The expression levels of phospho-CaMKII and phospho-RyR2 in the absence or presence of AhR antagonists, α-NF (10 nM) and propranolol (10 μM), were analyzed by western blotting (*n* = 5). The protein levels were normalized against those of β-actin levels. **d** The expression of SERCA2a in the absence or presence of α-NF were analyzed by western blotting (*n* = 5). Protein levels were normalized against those of β-actin. All values are represented as mean ± SD. *P < 0.05 or **P < 0.01 compared with control

The aryl hydrocarbon receptor (AhR) is a ligand-activated transcription factor that regulates biological responses to planar aromatic hydrocarbons and is known to act primarily as a sensor of xenobiotic chemicals [25, 26]. As shown in Fig. 6a, AhR translocated into the nucleus from the membrane in a time-dependent manner in cardiomyocytes treated with both forms of PAH. The amount of AhR translocated was significantly higher in cardiomyocytes treated with oxy-PAHs than in those treated with PAHs. In order to investigate whether the intracellular translocation of PAHs by AhR promotes calcium perturbation, we used AhR antagonists, α-naphthoflavone (α-NF) or propranolol to block the cellular translocation of AhR. We observed that AhR translocation was successfully inhibited by α-NF (Fig. 6b). Subsequently, decreased levels of phosphorylated CaMKII and RyR2 and increased SERCA2a levels were successfully rescued by treatment with α-NF or propranolol (Fig. 6c and d).

## Discussion

Several studies have shown that there is an association between ambient air particles and cardiovascular dysfunction; however, the underlying mechanisms are complex and variable and remain to be elucidated [27]. In particular, the effects on the heart are acute and, therefore, frequently lethal, making it imperative that their mechanisms in the myocardium are identified. The present study demonstrated that PM exposure significantly increases ROS generation and calcium perturbation, leading to electrophysiological instability in cardiomyocytes. In addition, results obtained suggest that these instabilities are mainly induced by the oxy-PAHs contained in PM. Outcomes from chemical intervention by NAC support a role for ROS in mediating these effects. In the present study, significant electrophysiological alterations were observed a few minutes after PM exposure; they were greater in cardiomyocytes treated with WPM than in those treated with SPM and were markedly attenuated by the ROS scavenger, NAC. These results suggest that electrophysiological changes in cardiomyocytes are primarily mediated by ROS generation.

Even though the mechanisms by which air pollutants influence the risk of cardiovascular events are still under investigation, there are several plausible theories [28]. After PM penetration beyond the upper respiratory tract into the parenchymal region of the lung [29], the lung releases pro-oxidative (i.e., ROS) and proinflammatory (i.e., cytokines, such as IL-6 and TNF-α) mediator and vasoactive hormones, such as endothelins, both locally and into the systemic circulation [30–32]. These secreted molecules could be related to PM-induced alterations during the autonomic control of the heart [2, 33], which are responsible for the occurrence of cardiovascular disease, especially arrhythmias. Indeed, some studies have shown that animals exposed to diesel exhaustion had reduced heart rate variability [34], and these experimental data are supported by several clinical studies that show a proportional relationship between PM concentration and heart rate variability [35, 36]. Decreased heart rate variability indicates the existence of a state of cardiac autonomy dysfunction and is an obvious risk factor for sudden cardiac death due to arrhythmias [37]. The underlying mechanisms responsible for electrophysiological alterations remain unclear but might involve direct effects of PM or indirect effects of biochemical molecules secreted by PM on cardiac ion channels [38].

Ambient particulate matter is a complex mixture containing various types of organic matter, PAHs, and inorganic metals. Indeed, it has been known that the metal-mediated generation of ROS can cause severe oxidative stress within cells or tissues through the oxidation of nucleic acids, proteins, and lipids [39, 40]. However, the main finding of this study is that arrhythmic parameters, such as resting membrane potential and amplitude, were significantly altered by PM treatment, and the degree of alteration was greater in WPM-treated cells, which contain more PAHs including oxy-PAHs. Furthermore, our results support the hypothesis that oxy-PAHs are more closely associated with a risk of cardiac arrhythmia than PAHs, because of the differential ROS generation. Indeed, there are some reports that the cardiac effects by DEP, especially arrhythmia, have been attributed to changes in autonomic activity that was not present in cells [41]. However, there are other recent data supporting our results that PM can cross into the pulmonary and systemic circulations directly affecting the heart and blood vessels [42] and DEP has shown both direct and indirect effects on cardiomyocyte functions [43]. The electrophysiological instability by PM or PAHs was completely blocked by pretreatment of the cells with an ROS scavenger, which is consistent with a recent study that revealed that the arrhythmogenic effects induced by DEP were prevented by antioxidant treatment and a CaMKII blockade [9]. In addition, we showed that PM treatment subsequently disturbed calcium homeostasis in cardiomyocytes. The expression levels of representative calcium regulating proteins, such as CaMKII and RyR2, were significantly altered. Interestingly, SERCA2a expression significantly decreased only in WPM-treated cardiomyocytes, and the levels of NCX2 were higher in cardiomyocytes treated with WPM than in those treated with SPM. These results suggest that the specific components or their concentrations in WPM affect cardiomyocyte redox stress and calcium perturbation more than the components of SPM, which is consistent with the result that PAHs increase intracellular calcium in various cell types in a dose-dependent manner [44].

The main composition of PAHs, including oxy-PAHs in sampled PM, was determined in a previous study. However, it is not clear which constituents contributed to the observed adverse effects, as there is complexity of components and many unverified molecules, such as a variety of metals, biological compounds, and elemental carbons [12]. In addition, although there is accumulating evidence that PAHs play a critical role in the production of oxidative stress, the differential effects between PAHs and oxy-PAHs have not been determined. The findings of this study show that exposure to oxy-PAHs induces more ROS and, subsequently, more electrophysiological instability than PAHs in cardiomyocytes. In addition, the effect on SERCA2a and RyR2 levels was significantly greater in cardiomyocytes treated with oxy-PAHs than in those treated with PAHs, and the effects were suppressed by the ROS scavenger NAC.

As mentioned above, we analyzed the concentrations of other various constituents including organic carbons (OC), elemental carbons (EC), dicarboxylic acids, and metals in seasonal PM10 samples and found that the concentrations of these constituents were not displying any significant differences between SPM and WPM (data not shown). Therefore, we concluded that the differentially induced electrophysiological instability by PM was mainly through PAHs, especially oxy-PAHs, in winter samples. Furthermore, oxy-PAHs entered the cells more rapidly than PAHs, then these molecules translocated into the nucleus through AhR. Also, the expression levels of AhR is about 2–10 fold higher in cardiac than in other tissues of mice; subsequently, expression of the target gene, CYP1A1, increased by ~ 10 fold in cardiac tissues compared to that in other tissues in BaP-treated mice [45]. This demonstrates that the electrophysiological instability induced by oxy-PAHs might be specific to the myocardium. Our results concur with previous results which showed that BaP enters cells via AhR, resulting in redox stress and c-Ha-ras activation in vascular smooth muscle cells, which was prevented by the ROS scavenger NAC [46]. However, the redox stress between BaP and BaP-3,6-quinone (BaPQ), an oxygenated derivative of BaP, was not evaluated in this study. Our result are also supported by several previous reports that 9,10-phenanthrenequinone, one of the major components of PM can cause an impairment of endothelium-dependent vasorelaxation through the regulation of eNOS activity and are associated with cardiopulmonary diseases [47, 48].

The present study has some methodological limitations. One of the drawbacks is the direct effect of PAHs on electrophysiological instability was revealed in cells rather than in whole heart or animal models. Therefore, the consequences of PM or PAHs treatment reported herein may not manifest in humans after real-world inhalation. Secondly, the concentrations of individual PAHs used in this study was slightly higher than the concentration of mobilized PAHs from PM. Although there is a previously reported association between high-pollution days and the increased incidence of acute cardiovascular events [49], further investigation for the association between the mobilized constituents and their concentration and induction of electrophysiological instability will be needed. Even though there are limitations as shown above for this study, our results of the effects of PAHs, especially oxy-PAHs, on electrophysiological instability in cardiomyocytes might be expanded to the area of mammalian cardiotoxicity. Indeed, high-throughput in in vitro cardiotoxicity of 69 environmental chemicals was successfully assessed using human induced pluripotent stem cell-derived cardiomyocytes [50].

## Conclusions

Our results provide strong evidence that ambient PM increases arrhythmia by ROS generation, and oxy-PAHs are the key components of PM in this regard. Electrophysiological instability and subsequent calcium perturbation by PM or PAHs were successfully attenuated by an ROS scavenger. The adverse effects of oxy-PAHs, which are mediated by AhR, are more severe than those of PAHs. AhR is highly abundant in cardiac tissue, making the arrhythmogenicity of oxy-PAHs particularly hazardous. Although there is an increasing amount of clinical evidence supporting our findings of cardiac electrophysiological instability by ambient PM, the in vivo and clinical relevance of these findings further remains to be elucidated.

## Methods

### Ambient particulate matters and preparation of organic components

Collection of ambient particulate matter (PM) at the Seoul metropolitan area and particle preparation was described in a previous study [51] which showed the detailed process for sampling of PM10 and extraction of organic matter from PM. Briefly, PM10 samples were collected at the roof of a public health building of Seoul National University in the Seoul metropolitan area. The sampling site is surrounded by commercial and residential areas of the city. PM10 samples were collected on quartz fiber filters (QFFs) (20.3 × 25.4 cm^2^) for 24 h in summer (June–August) and winter (December–February). After sampling, the filter was wrapped in pre-baked aluminum foils and stored in a freezer (− 20 °C) until analysis. The filter was extracted by sonication with a mixture of dichloromethane (DCM) and methanol (3:1, *v/v*) for 30 min. Extracts were evaporated under a stream of N_2_ gas (Zymark Turbo Vap II) down to a volume of 10 mL and then was filtered using a 0.45 μm syringe filter. For further usage, the extracts were diluted 10-fold with dimethylsulfoxide (DMSO). The analyzed organic compounds including PAHs and oxy-PAHs and their concentrations were presented in Table 1.

### Cell culture and treatments

Neonatal rat cardiomyocytes were isolated and purified by modifying previously described methods [52]. Briefly, 2–3 day old Sprague-Dawley rat pups were disinfected with povidone and then dissected. The chests of these rats were opened and their hearts were rapidly removed and washed with the Phosphate-buffered saline solution (pH 7.2, WelGENE) lacking Ca^2+^ and Mg^2+^. Using micro-dissecting scissors, hearts were minced until the pieces were approximately 1 mm^3^ and treated with 5 mL of collagenase type II (0.9 mg/mL, 210 units/mg, Gibco BRL) for 7 min at room temperature. The cells in the supernatant were transferred to a tube containing cell culture medium (α-MEM containing 10% fetal bovine serum, WelGENE). The tubes were centrifuged at 1200 rpm for 4 min at room temperature, and the cell pellets were resuspended in 3 mL of cell culture medium. The above procedures were repeated 6–8 times until only a little tissue was left. Cell suspensions were washed twice with cell culture medium and seeded to achieve a final concentration of 5 × 10^5^ cells/mL and then they were plated onto gelatin-coated 6-well plates. The cells were cultured in α-MEM containing 10% fetal bovine serum with 0.1 mM bromodeoxyuridine (BrdU), which was used to prevent proliferation of cardiac fibroblasts. Cells were then cultured in 5% CO_2_ incubator at 37 °C. The cells were then treated with designated volumes of PM extracts mentioned above and DMSO (0.1% of final concentration) is used as a control treatment.

### Patch-clamp recordings

The cells were bathed in external solution containing (mM): NaCl 135, KCl 5.4, MgCl_2_ 1.0, CaCl_2_ 1.8, NaH_2_PO_4_ 0.33, glucose 5, and HEPES 10 and was adjusted to pH 7.4 with Tris buffer. The pipetted solution contained (in mM): Mg-ATP 3, CsCl 140, HEPES 10 and EGTA 10 and was adjusted to pH 7.2 with Tris buffer. Currents or potentials were amplified using an Axopatch 200B (Axon Instruments) and digitized with a 16-bit analog to digital converter (Digidata 1550A; Axon Instruments). The data were filtered at 5 kHz and was displayed on a computer monitor. Results were analyzed using pClamp software (version 9.2; Axon Instruments) and GraphPad Prism software. All experiments were performed at 30 °C.

### Measurement of intracellular reactive oxygen species (ROS)

Intracellular ROS were measured using a fluorescent dye technique. Cardiomyocytes were seeded onto a 24-well plate with glass cover slips at a density of 5 × 10^4^ cells/mL and cultured for 24 h. Then, the cells were treated with a negative control (DMSO), positive control (200 nM of H_2_O_2_), SPM, and WPM in a dose dependent manner for 1 h. Then, the cells were washed twice with calcium free PBS (PBSc) and loaded with 2′,7′-dichlorofluorescin diacetate (H_2_DCF-DA, Invitrogen, USA) and 4′,6-diamidino-2-phenylindole (DAPI) diluted with calcium free warm PBS to a final concentration of 10 μM and 50 μg/mL, respectively. Then, the cells were incubated for 10 min at 37 °C in the dark. The probe H_2_DCF-DA (10 μM) entered into the cells, and the acetate groups on H_2_DCF-DA were cleaved by cellular esterases, trapping the nonfluorescent 2′,7′-dichlorofluorescin (DCFH) within the cells. Subsequent oxidation by reactive oxygen species yielded a fluorescent product DCF. Then, the cells were gently washed under the coverslips three times in warm PBS and the coverslips were placed in the chamber, which was mounted on the stage of an inverted microscope equipped with a confocal laser-scanning system. The dye, when exposed to an excitation wavelength of 480 nm, emitted light at 535 nm only when it had been oxidized. Fluorescence images were collected using a confocal microscope (Fluoview FV1000 confocal system, Olympus) by excitation at 488 nm and emission greater than 500 nm with a long-pass barrier filter. The fluorescence intensity of an equivalent field size (3 × 3 mm) in a plate was measured using Image J quantification software.

### Measurement of intracellular calcium levels

The intracellular calcium was measured using a fluorescent calcium indicator, Fluo-4 AM (Invitrogen). Cardiomyocytes were seeded onto a 4-well chamber at a density of 1 × 10^5^ cells/mL and cultured for 24 h. Then, the cells were treated a negative control (DMSO), SPM, and WPM in a dose dependent manner for 20 min. The cells were then washed with a serum free medium (α-MEM, WelGENE) and loaded with Fluo-4 AM diluted with serum free medium to a final concentration of 2 μM and incubated for 20 min at 37 °C in the dark. Then, the cells were washed twice with warm PBS buffer and covered with a cover slip. Fluo-4 AM fluorescence imaging was performed using a confocal microscope (Fluoview FV1000 confocal system, Olympus). Fluo-4 AM was excited with the laser at 488 nm, and fluorescence was measured at a wavelength of 515 nm.

### Immunocytochemistry

Cardiomyocytes were cultured onto a 24-well plate with glass cover slips at a density of 5 × 10^4^ cells/well. The cells were then fixed with 4% paraformaldehyde for 20 min and quenched with 1 M ethanolamine diluted in PBSc. After washing, cells were blocked with 0.5% bovine serum albumin in PBS for 30 min, then the blocking solution was removed and the cells were incubated overnight at 4 °C with rabbit anti-AhR (1:100 dilutions, BioWorld). Cells were washed and incubated with mouse anti-rabbit IgG-TR (1:1000 dilutions, Santa Cruz Biotechnology) at room temperature for 1 h. Then, the cells were gently washed under the cover slip three times with PBS and visualized under a laser scanning confocal microscope (Fluoview FV1000 confocal system, Olympus).

### Protein carbonylation colorimetric assay

Cardiomyocytes were exposed to PAH and oxy-PAH for 24 h. Each serum was centrifuged at 14,000 rpm for 10 min to eliminate all particulate matter that might interfere with this reaction. Then, a solution of 10 mM 2,4-dinitrophenylhydrazine (DNPH) in 2 N HCl was added to the serum containing protein (1 mg/mL) of each sample, incubate for 45 min at room temperature in the dark with occasional mixing. A blank reagent protein sample that reacted with 2 N HCl was added to each sample. Then, with 20% trichloroacetic acid (TCA) was added to each samples and centrifuged for 10 min on ice. The supernatants was discarded, and protein pellets were washed 5 times with 1 mL of ethanol/ethyl acetate (1:1, *v/v*) to remove any free DNPH. After the final washing step, samples were resuspended in 6 M guanidine hydrochloride, which is a protein solubilization solution, and vortexed thoroughly and incubated at 37 °C for 10 min. Then, the samples were centrifuged at 14,000 rpm for 10 min to remove any debris. To determine the protein concentrations of the solubilized protein sample, Bradford protein assay (Bio-Rad, Hercules) was performed. Carbonyl contents are determined from the absorbance measured at 375 nm against the blank for each sample using a molar absorption coefficient of 22,000 M^− 1^ cm^− 1^.

### Lipid peroxidation (MDA) assay

The amount of lipid peroxidation was estimated by measuring the amounts of thiobarbituric acid-reactive substances (TBARS). Briefly, samples were incubated with 0.5% TBA in 20% acetic acid solution (pH 3.7). After incubation at 95 °C for 40 min, the samples were kept on ice, and then centrifuged at 4000 rpm for 10 min. TBARS contents were determined by measuring absorbance at 532 nm. TBARS values were calculated by using a malondialdehyde (MDA) standard curve. Results were expressed as nmol MDA/mg protein.

### Cardiomyocyte microsomes preparation and Ca^2+^-ATPase activity assay

Cardiomyocytes were harvested, washed twice in 0.9% NaCl. Then, the cells were resuspended and incubated with lysis buffer (10 mM Tris, pH 7.5 and 0.5 mM MgCl_2_) on ice for 10 min and then 0.1 mM phenylmethanesulfonylfluoride (PMSF) was added. After lysis, the cells were homogenized with a disposable homogenizer (BioMasher), and then a solution containing 0.5 M sucrose, 10 mM Tris (pH 7.5), 40 μM CaCl_2_, 6 mM β-ME and 0.3 M KCl was added, and the cells were homogenized for an additional lysis step. The cell homogenate was then centrifuged at 14,000 rpm for 20 min. The supernatant solutions were then transferred to another ultracentrifuge tube containing 2.5 M KCl and centrifuged at 90,000 rpm for 1 h. The pellets were washed and resuspended with wash buffer (0.25 M sucrose, 10 mM Tris (pH 7.5), 20 μM CaCl_2_, 3 mM β-ME, 0.15 M KCl), and the protein concentrations were determined using Bradford protein assay. Ca^2+^-ATPase activity was determined by measuring the quantity of inorganic phosphate (Pi) liberated from the hydrolysis of ATP by colorimetric assay. The microsome membranes (SERCA2a 30 μg/mL) were incubated with the reaction buffer (50 mM MOPS, 100 mM KCl, 5 mM MgCl_2_, NaN_3_, 1 mM EGTA and 1 mM CaCl_2_ pH 7.0) for 10 min, and 10 mM ATP (final concentration, 1 mM) was added. After 30 min of incubation, the reaction mixture was measured by a Malachite green phosphate assay kit (BioAssay Systems). The absorbance of the resulting colored complex was determined at 620 nm. The quantity of Pi was calculated by using a phosphate (KH_2_PO_4_) standard curve.

### Real-time quantitative PCR (qPCR)

The expression levels of various genes were analyzed by a qPCR assay. The cells were seeded into a 6-well plate with glass cover slips at a density of 5 × 10^5^ cells/mL and cultured for 24 h. The cells were treated with either negative control (DMSO), SPM, or WPM in a dose dependent manner for 12 h. Total RNA was extracted using TRIzol lysis reagent (QIAGEN) according to the instructions provided by the manufacturer. The total RNA concentration of each sample was measured by a spectrophotometer (Eppendorf) at 260 nm. Total RNA was subjected to reverse transcription using HelixCript™ 1st-Strand cDNA Synthesis Kit (NanoHelix). Real-time quantitative PCR with realHelix™ qPCR kit (NanoHelix) was performed by the SYBR Green method using an Applied Rotor-Gene 3000™. Gene expression was normalized to GAPDH. The relative mRNA expression levels were quantified and analyzed using Rotor-Gene 6 software (Corbett-research) using ^△△^Ct methods. Table 3 shows all the primer sequences used for qPCR.

**Table 3:** Sequences of primers used for real-time quantitative PCR

Gene	Primer sequence
GAPDH	Sense: 5′-CAGTGCCAGCCTCGTCTCAT-3′ Antisense: 5′-TGGTAACCAGGCGTCCGATA-3’
SERCA2a	Sense: 5’-CGAGTTGAACCTTCCCACAA-3′ Antisense: 5′-AGGAGATGAGGTAGCCGATGAA-3’
RyR2	Sense: 5’-CAAACAGGGCAGAAGACACC-3′ Antisense: 5′-CTCTGAGGGTGCTCCACCT-3’
CaMKII	Sense: 5’-CATCCTGAACCCTCACATCCA-3′ Antisense: 5′-CCGCATCCAGGTACTGAGAGTGAT-3’
Calsequestrin2	Sense: 5’-TCAAAGACCCACCCTACGTC-3′ Antisense: 5′-AGTCGTCTGGGTCAATCCAC-3’
CalcineurinA	Sense: 5’-TGGTGAAAGCCGTTCCATTT-3′ Antisense: 5’ CCCATCGTTATCAAACACTTCCT-3’
Calmodulin	Sense: 5′-GGCATCCTGCTTTAGCCTGAG-3′ Antisense: 5′-ACATGCTATCCCTCTCGTGTGAC-3’
NCX1	Sense: 5’-AGCAAGGCGGCTTCTCTTTT-3′ Antisense: 5′-GCTGGTCTGTCTCCTTCATGT-3’
NCX2	Sense: 5’-CACTACGAGGATGCTTGTGG-3′ Antisense: 5′-CCTTCTTCTCATACTCTTCGT-3’
NCX3	Sense: 5’-CCTGTGGCTCCTCTACGTACTCTT-3′ Antisense: 5′-GAGGTCTTGTTCTGGTGGTTCA-3’

### Immunoblot analysis

Cardiomyocytes were seeded onto a 6-well plate at a density of 5 × 10^5^ cells/mL and cultured for 24 h. Cells were then treated with either negative control (DMSO), PAHs, or oxy-PAHs in dose dependent manner for 24 h. The cells were then washed once in PBS buffer and lysed in RIPA buffer containing PMSF and phosphatase inhibitor. The protein concentrations were determined using the Bradford protein Assay. Proteins were separated in a 6–10% sodium dodecyl sulfate-polyacrylamide gel and transferred to a polyvinylidiene difluoride membrane (Bio-Rad laboratories, Inc.). After blocking the membranes with Tris-buffered saline-Tween 20 (TBS-T, 0.1% Tween 20) containing 5% skim milk for 1 h at room temperature, the membranes were incubated with a primary antibody for overnight at 4 °C. The primary antibodies were used at the following dilutions in blocking buffer: phospho Akt (1:200, #9271, Cell Signaling Technology), Akt (1:1000, #9297, Cell Signaling Technology), phospho ERK (1:1000, #9101, Cell Signaling Technology), ERK (1:1000, SC-135900, Santa Cruz), β-actin (1:5000, Sigma), CaMKll (1:500, LF-PA20064, AbFRONTIER), p-CaMKll (1:500, LF-PA20065, AbFRONTIER), ATP2A2/SERCA2 (1:5000, Cell Signaling Technology), RYR2 (1:500, 19,765–1-AP, Proteintech Group) and p-S2808 RYR2 (1:500, ab59225, Abcam). The membrane was washed five times with TBS-T for 5 min and incubated for 1 h at room temperature with secondary antibodies. After extensive washing, bands were detected by an enhanced chemiluminescence reagent (ECL, BIONOTE, Animal Genetics Inc.). The band intensities were quantified using the Image J quantification software.

### Statistical analysis

All quantified data from at least triplicate measurements were analyzed with SPSS 13.0 software. Data are expressed as mean ± SD. Statistical comparisons between two groups were performed using the Student’s t-test. Statistical comparisons among multiple groups were performed using analysis of variance (ANOVA). A two-tailed *P* < 0.05 was considered statistically significant.

## Data Availability

The datasets used and/or analyzed during the current study are available from the corresponding author on reasonable request.
